# Emotion regulation choice: selecting between cognitive regulation strategies to control emotion

**DOI:** 10.3389/fnhum.2013.00179

**Published:** 2013-05-08

**Authors:** Gal Sheppes, Ziv Levin

**Affiliations:** The School of Psychological Sciences/Child Clinical, Tel Aviv UniversityTel Aviv, Israel

Consider the anger that arises in a heated argument with your romantic partner, or the dreadful anxious anticipation in the dentist's waiting room prior to a root canal procedure. Our daily lives are densely populated with events that make us emotional. Luckily, however, we developed numerous ways to control or regulate our emotions in order to adapt (Gross, 2007; Koole, 2009 for reviews). A central remaining challenge to explain adaptation, involves understanding how individuals choose between the different emotion regulation strategies in order to fit with differing situational demands. Specifically, when is the aforementioned romantic partner or dental patient more likely to “put aside” or disengage from the emotional situation, and when are they more likely to “make sense” or engage with their emotional reactions?

In this opinion article we concentrate on the intersection between affective science and decision making as manifested in emotion regulation choice, defined as the act of making an autonomous choice between different regulation strategies that are available in a particular context.

## How important are our emotion regulation choices?

Recent advances in the field of emotion regulation suggest that regulation strategies have different consequences in different contexts. Accordingly, several emerging conceptual accounts emphasize the importance of *flexibly choosing* between emotion regulation strategies in a manner that is adaptive to differing situational demands (e.g., Bonanno, 2005; Kashdan and Rottenberg, 2010; Troy and Mauss, 2011; for reviews).

While emotion regulation choice has become an important concept in modern conceptual accounts, direct empirical support has been lacking until recently. The main reason is that previous experimental studies in the field have instructed participants to employ rather than choose between different regulation strategies (e.g., Bonanno et al., 2004; Westphal et al., 2010; Webb et al., 2012, for a recent review), leaving the determinants and underlying mechanisms of emotion regulation choice unexplored.

To address these important gaps we recently developed a conceptual framework to explain the (1) major determinants and (2) underlying mechanisms of emotion regulation choice (Sheppes et al., 2011, 2013; Sheppes, in press). The starting point of this conceptual framework was set to explain the differential consequences of employing (rather than choosing between) different regulation strategies (Sheppes and Gross, 2011, 2012). Specifically, according to this framework due to limited cognitive capacity, a constant competition emerges between emotion generation and emotion regulation processes (Gross et al., 2011a,b) for dominance over behavior. The conceptual account borrows from information processing theories (e.g., Pashler, 1998; Hubner et al., 2010) and the process model of emotion regulation (Gross and Thompson, 2007) to suggest that emotion regulation, involves recruiting *deliberate executive control mechanisms* that try to modify the nature of emotional information processing at two major cognitive stages: early attentional selection and late semantic meaning stages.

Incoming emotional information can be regulated at an early attentional selection processing stage by *disengaging* from emotional information processing before it undergoes elaborated processing in working memory (the aforementioned “put aside” option). A classic early selection strategy is distraction, which involves producing neutral thoughts that are independent from and not in conflict with emotional information (e.g., van Dillen and Koole, 2007; Thiruchselvam et al., 2011). Engagement with incoming emotional information that passes the early attentional selection stage can still be regulated at a late semantic meaning processing stage before it determines behavior (the aforementioned “make sense” option). A classic late selection regulation strategy is reappraisal, which involves changing the meaning of emotional information in a late processing stage (e.g., Gross, 2007; Thiruchselvam et al., 2011; Blechert et al., 2012). In reappraisal, the original emotional appraisal functions as the building block of the reinterpretation, and as such the two are semantically dependent and in direct conflict.

According to the conceptual framework, the underlying characteristics of disengagement distraction and engagement reappraisal result in a differential cost-benefit tradeoff (Sheppes and Gross, 2011, 2012). Specifically, *emotionally* blocking affective information early before it gathers force via distraction can modulate high intensity information more successfully, relative to reappraisal that allows emotional information to gather force prior to a late modulation (Sheppes and Meiran, 2007). *Cognitively*, the generation process in distraction that involves producing neutral thoughts that are independent from and not in conflict with the original emotional information, is simpler than generating reappraisals, where neutral reinterpretations are in direct conflict with emotional appraisals (Sheppes and Meiran, 2008; Sheppes et al., 2009). *Motivationally*, distraction does not allow for emotional events to be attended to and provided with adequate explanation which is non-beneficial in many emotional events where long term adaptation requires facing difficulties in order to adapt (Wilson and Gilbert, 2008 for a review), relative to reappraisal which allows emotional processing (Kross and Ayduk, 2008; MacNamara et al., 2011; Thiruchselvam et al., 2011; Blechert et al., 2012).

Utilizing the original framework to explain emotion regulation choice involved hypothesizing that regulatory choices of healthy individuals would be sensitive to the costs and benefits tradeoff associated with the implementation of each regulatory option in different contexts. With regard to underlying mechanisms of emotion regulation choice we argue that healthy regulation choice requires, in some contexts, the ability to recruit deliberate executive control processes that can override contrasting associative emotional processes (cf. Muraven and Baumeister, 2000). Moreover, differences in strategies' underlying engagement with or disengagement from emotional processing dimension heavily determine regulation choice, relative to other potent factors such as differential cognitive effort.

## Emotion regulation choice: emotional, cognitive, and motivational determinants

The first determinant of regulation choice examined is *emotional* intensity which is a key dimension of variation across emotional contexts (Sheppes et al., 2011). To test our predictions, we manipulated emotional intensity with emotional images or unpredictable electric stimulation and had participants choose between distraction and reappraisal (Sheppes et al., 2011). Confirming the conceptual framework, we found that under low negative intensity situations, participants prefer late selection engagement reappraisal over early selection disengagement distraction, presumably because reappraisal can both successfully modulate immediate emotional responding as well as provide long term adaptation. However, under high negative intensity situations participants mostly prefer early disengagement distraction over reappraisal, because only distraction can successfully block emotional information before it gathers force. A follow up study demonstrated the robustness of this effect in showing that both regulatory preferences are maintained even when participants are offered high monetary amounts to choose the contrasting strategy (Sheppes et al., 2013).

The second determinant of regulation choice examined was the *cognitive* complexity of generating a strategy (Sheppes et al., 2013). According to the conceptual framework, the generation process in reappraisal is more complex than in distraction because the formation of a neutral reinterpretation depends on the original appraisal of emotional information. It was therefore predicted and found that when the generation process was simplified, by providing participants with concrete regulatory suggestions for distraction and reappraisal, reappraisal was more frequently chosen.

The third determinant of emotion regulation choice involved investigating the influence of *motivational* goals (Sheppes et al., 2013). According to our framework, emotional stimuli that are encountered multiple times can be better regulated for long term adaptation with strategies like reappraisal that involve engaging with emotional processing. As predicted, it was found that participants who anticipated encountering emotional stimuli more than once preferred to reappraise more than participants who expected to encounter each emotional stimulus only once.

The aforementioned emotional, cognitive, and motivational factors tended to independently influence regulatory choices between distraction and reappraisal manifested in findings main effects.

## Emotion regulation choice: underlying mechanisms

According to our conceptual framework, emotion regulation choice should involve a general ability of deliberate executive control processes to override competing associative emotional processes. An alternative more parsimonious account, suggests that emotion regulation choice can be fully explained by a direct influence from simple associative emotional processes (e.g., Bradley et al., 2001). Specifically, a basic defensive system directly motivates the organism toward engagement (resulting in reappraisal) under low negative intensity situations, and toward disengagement (resulting in distraction) under high negative intensity. To determine between the two accounts we investigated a context where the two accounts would diverge—down-regulation of positive-emotional situations. Specifically, the associative-emotional process account would argue that as positive emotional intensity increases it directly activates a basic appetitive system that would lead to an increased preference to engage. By contrast, we found that the operation of deliberate control processes, whose goal is to provide down-regulation of positive emotional situations, involved overriding the associative tendency to engage, resulting in an increased preference to disengage as positive emotional intensity increased (Sheppes et al., 2013).

A further investigation of underlying mechanisms involved asking what are the dimensions that receive central weight in the choice between distraction and reappraisal? Two potential central dimensions include engagement/disengagement and cognitive effort involved in distraction and reappraisal. Specifically, when people prefer to distract in high negative emotional intensity situations, are they choosing distraction mainly because they prefer to disengage from emotional processing or mainly because they prefer to reserve cognitive resources?

To begin investigating this issue we pitted these two alternative accounts by having participants choose between two types of distractions: one regulatory option was cognitively simple and involved minor disengagement from emotional processing (performing mathematical subtract 2s) and a second regulatory option was cognitively effortful yet highly disengaging from emotional processing (subtract 7s). Findings supported the centrality of the engagement/disengagement factor with an increased preference to use the more disengaging (despite it being also more effortful) subtract 7s distraction as negative emotional intensity increased. These findings suggest that individuals are willing to exert substantial cognitive effort in order to obtain adequate levels of disengagement. Nevertheless, future studies should parametrically manipulate varying levels of engagement/disengagement and cognitive effort in order to better understand the relationship between them. In a complementary study we showed that the engagement/disengagement dimension is central within the reappraisal category. Specifically, we found that under high negative emotional intensity participants choose to use “reality challenge” reappraisals (e.g., “this picture is fake”) which involves disengaging by not considering emotional consequences of events (Sheppes et al., 2013).

We end this section with broader considerations that should be investigated in future studies. First, while our conceptual model makes a broad distinction between early and late selection regulation strategies, empirical support comes from studies that concentrate on only one early selection strategy (distraction) and one late selection strategy (reappraisal). It is clear that people typically use many other strategies and that their regulatory choice patterns may have important consequences for well-being and psychopathology. Consider avoidance which disengages from emotional processing at an early selection stage, and rumination which involves magnifying emotional information at an early attentional stage and elaborating it in a late selection phase. Our account suggests that deviations from the preference to disengage from high emotional intensity by overly engaging via rumination may be related to depression (Nolen-Hoeksema et al., 2008). At the same time, deviations from the preference to engage with tolerable emotional intensity events by disengaging via avoidance may be linked to anxiety disorders (Campbel-Sills and Barlow, 2007).

Second, we concentrated on deliberate regulatory choices among explicit regulation strategies. While the vast majority of studies in the field concentrated on explicit forms of regulation (Gross and Thompson, 2007), implicit forms of emotion regulation are central and dominant (Gyurak et al., 2011). Given that unconscious processes can perform most complex functions (Hassin, 2013), it may well be that regulatory decision making processes, including those that make use of central executive resources (see Marien et al., 2012), can be performed unconsciously. Central factors such as prior practice with choosing regulation strategies in different situations, strong motivational forces to perform one strategy over another and a general central executive ability that allows efficient information processing may all influence regulatory choices. Future studies should link explicit and implicit processes in determining emotion regulation choice (see Sheppes and Gross, under review, for such an effort).
